# Comparison of the Effects of Sodium-Glucose Cotransporter 2 Inhibitors on Cardiac Fibroblast Properties

**DOI:** 10.3390/ijms262010098

**Published:** 2025-10-16

**Authors:** Claire Baufays, Julien Cumps, Cécile Dufeys, Audrey Ginion, Luc Bertrand, Sandrine Horman, Christophe Beauloye, Alice Marino

**Affiliations:** 1 Pôle de Recherche Cardiovasculaire, Institut de Recherche Expérimentale et Clinique, Université Catholique de Louvain, 1200 Brussels, Belgium; claire.baufays@uclouvain.be (C.B.); julien.cumps@uclouvain.be (J.C.); cecile.dufeys@uclouvain.be (C.D.); audrey.ginion@uclouvain.be (A.G.); luc.bertrand@uclouvain.be (L.B.); sandrine.horman@uclouvain.be (S.H.); 2 Division of Cardiology, Cliniques Universitaires Saint-Luc, 1200 Brussels, Belgium; 3 Division of Cardiovascular Intensive Care, Cliniques Universitaires Saint-Luc, 1200 Brussels, Belgium

**Keywords:** cardiac fibroblasts, sodium-glucose cotransporter 2 inhibitors, AMPK

## Abstract

Recent clinical trials have shown significant cardioprotective effects of antidiabetic sodium-glucose cotransporter 2 inhibitors (SGLT2i), including canagliflozin, empagliflozin, and dapagliflozin. These drugs significantly reduce hospitalizations for heart failure with reduced and preserved ejection fraction in both diabetic and non-diabetic patients. Yet, the mechanisms underlying their protective effects, beyond their glucose-lowering properties, remain poorly understood. This study aimed to elucidate the direct effects of SGLT2i on cardiac fibroblasts, key mediators of myocardial fibrosis, ventricular remodeling, and heart failure. Using primary human cardiac fibroblast cultures, we compared the impact of canagliflozin, empagliflozin, and dapagliflozin on fibroblast properties. All three inhibitors significantly prevented myofibroblast differentiation. Notably, only canagliflozin significantly reduced fibroblast proliferation and migration. While all SGLT2i increased AMP-activated protein kinase (AMPK) phosphorylation, their effects on myodifferentiation were AMPK-independent. In contrast, the effect of canagliflozin on migration was partially dependent on AMPK, as demonstrated using the AMPK inhibitor BAY-3827. These findings reveal distinct cellular effects of individual SGLT2i on cardiac fibroblasts, suggesting heterogeneous potential to modulate extracellular matrix remodeling. Among them, canagliflozin may be more potent in preventing myocardial fibrosis in the context of heart failure.

## 1. Introduction

Sodium-glucose cotransporter (SGLT) 2 inhibitors (SGLT2i), also known as gliflozins, are antidiabetic drugs that block the SGLT2 cotransporter located in the proximal tubule of the kidney responsible for approximately 90% of filtered glucose reabsorption [1]. The resulting increase in urinary glucose excretion leads to reduced fasting and postprandial blood glucose levels, independently of insulin secretion. Several SGLT2i, including canagliflozin, empagliflozin, and dapagliflozin, derive from phlorizin, an early non-selective SGLT inhibitor isolated in 1835 from the root bark of the apple tree [2,3]. Although all are gliflozins, they differ in half-lives and selectivity for SGLT2 over other SGLTs [4,5].

Clinical trials in type 2 diabetes patients have shown that SGLT2i not only satisfy safety criteria but also significantly reduce hospitalizations for heart failure and cardiovascular mortality [6,7,8]. Subsequent cardiovascular outcome trials have confirmed that these benefits extend to patients with established heart failure, with both reduced or preserved ejection fraction, and regardless of their diabetic status [9,10,11,12]. SGLT2 expression is not detectable in the healthy heart [13,14,15], leaving the precise mechanisms underlying their cardioprotective effects largely unresolved [16,17].

Accordingly, extensive research has focused on uncovering the mechanisms underlying these effects in the failing heart, revealing several SGLT2-independent actions in cardiac cells. For example, SGLT2i improve cardiac function by directly inhibiting the cardiac sodium hydrogen exchanger (NHE) [18,19,20,21,22], by reducing reactive oxygen species (ROS) [23,24,25,26,27,28] and attenuating inflammation [29,30], while also improving glucose uptake and insulin sensitivity. However, beyond their direct actions on cardiomyocytes, gliflozins also appear to exert anti-fibrotic effects. Numerous studies have reported that SGLT2i also reduce cardiac fibrosis, a critical determinant of adverse ventricular remodeling and heart failure progression, suggesting their potential action on cardiac fibroblasts. While collagen deposition is essential for tissue repair following injury, its excessive accumulation promotes myocardial stiffness and increases the risk of cardiovascular events [31,32]. In rodent models of heart failure, SGLT2i have been shown to suppress collagen synthesis and fibroblast activation, primarily via modulation of the AMPKα/TGF-β/Smad signaling, with AMPK acting as a key energy sensor and regulator of fibrotic responses [29,33,34,35,36]. However, the direct effects of gliflozins on cardiac fibroblast function remains poorly characterized. Hence, we hypothesized that SGLT2i directly modulate cardiac fibroblast activation through AMPK-dependent mechanisms, thereby attenuating fibrosis, myocardial stiffness, and dysfunction. Building on our previous work showing that AMPKα1 regulates cardiac fibroblast activation and proliferation post-myocardial infarction [37,38], we investigated and compared the effects of canagliflozin, empagliflozin, and dapagliflozin on cardiac fibroblast myodifferentiation, proliferation, and migration using cultured human cardiac fibroblasts (HCF). This study identifies both AMPK-dependent and -independent mechanisms by which SGLT2i directly influence cardiac fibroblast activity and contribute to myocardial remodeling.

## 2. Results

### 2.1. Differential Effects of Canagliflozin, Empagliflozin, and Dapagliflozin on Human Cardiac Fibroblast Properties

**Figure 1:**
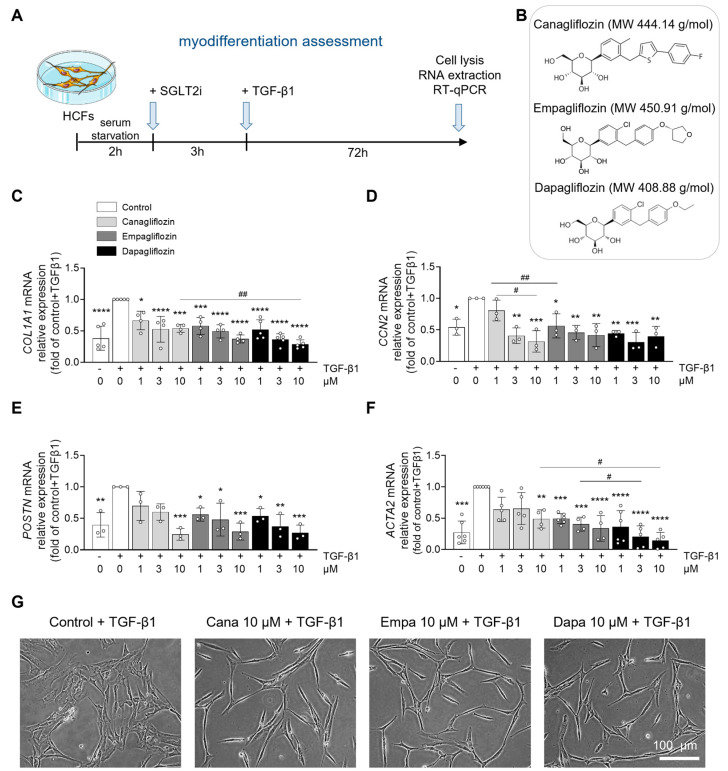
**Canagliflozin, empagliflozin, and dapagliflozin prevent cardiac fibroblast myodifferentiation.** (**A**) Schematic representation of the experimental protocol. HCFs were treated with DMSO (vehicle) or indicated concentrations of SGLT2i for 72 h. TGF-β1 (10 ng/mL) was added 3 h after SGLT2i to induce HCF myodifferentiation. (**B**) Chemical structures and molecular weight (MW) of canagliflozin, empagliflozin, and dapagliflozin. mRNA expression levels of the fibrotic markers (**C**) *COL1A1*, *CCN2* (**D**), and myodifferentiation markers (**E**) *POSTN* and (**F**) *α-SMA* were measured by RT-qPCR on RNA extracts. (**G**) Representative images (scale bar: 100 µm). Data are expressed as mean ± SD (*n* = 3 to 6 biological replicates for each condition). Cell density for each condition: 7500 c/cm^2^ (in 10 cm diameter dishes = 60.8 cm^2^). * *p* < 0.05, ** *p* < 0.01, *** *p* < 0.001. **** *p* < 0.0001 are relative to the TGF-β1-stimulated control condition. Statistical significance was determined by one-way ANOVA followed by Sidak’s multiple comparisons test. Statistical differences between different drug concentrations were performed by two-way ANOVA followed by multiple comparisons test. ^#^ *p* < 0.05, ^##^ *p* < 0.01 relative to different drugs concentrations.

**Figure 2:**
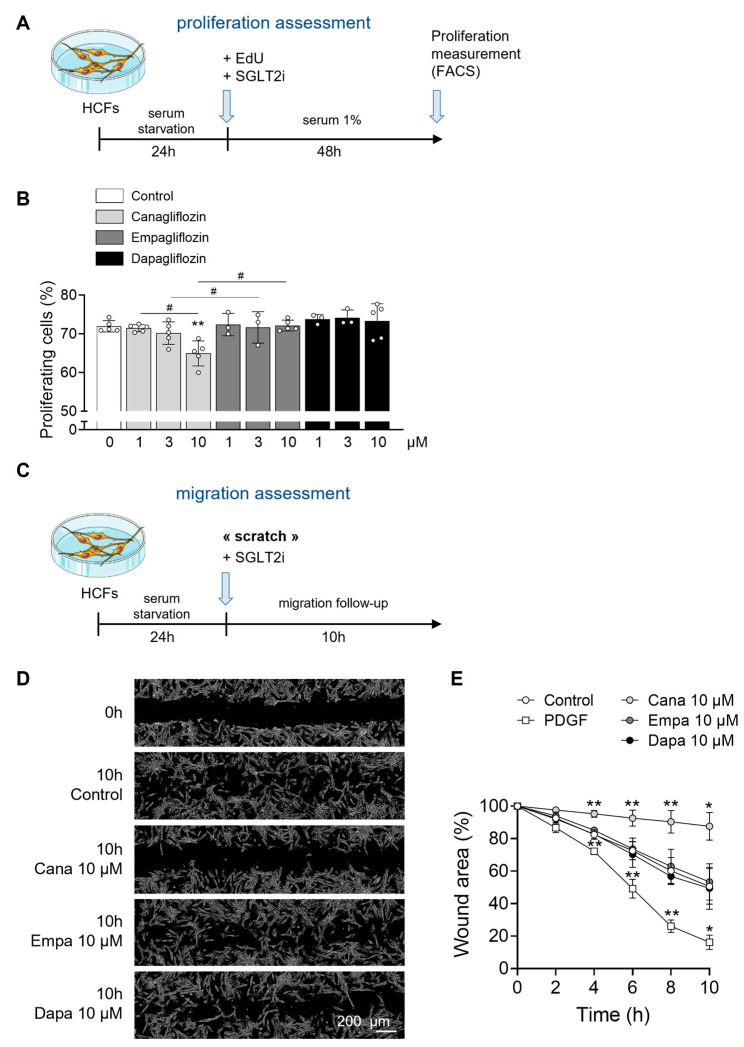
**Only canagliflozin inhibits cardiac fibroblast proliferation and migration.** (**A**) Schematic representation of proliferation assessment. HCFs were treated with DMSO (vehicle) or indicated concentrations of SGLT2i in a medium containing 1% growth factors for 48 h. Proliferation was quantified by flow cytometry using 5-ethynyl-2′-deoxyuridine (EdU). (**B**) Percentage of proliferating (EdU-positive) cells. Cell density for each condition: 5000 c/cm^2^ (in 6 cm diameter dishes = 21.5 cm^2^). (**C**) Schematic representation of migration assessment. A scratch was made in the cell layer and HCFs were treated with DMSO (vehicle), PDGF (40 ng/mL) used as a positive control, or indicated concentrations of SGLT2i. Microscopic images were taken at baseline and after 2, 4, 6, 8, and 10 h to assess the refilling of the wound by fibroblasts. (**D**) Representative images (scale 200 µm) and (**E**) quantification of wound recolonization. Cell density for each condition: 10 000 c/cm^2^ (in 3.5 cm diameter dishes = 9.4 cm^2^). For (**B**), data are expressed as mean ± SD (*n* = 3 to 5 biological replicates for each condition). ** *p* < 0.01 compared to the control condition. Statistical significance was determined by one-way ANOVA followed by Sidak’s multiple comparisons test. Statistical differences between different drugs concentration were performed by two-way ANOVA followed by multiple comparisons test. ^#^ *p* < 0.05 relative to different drugs concentrations. For (**E**), data are expressed as mean ± SD (*n* = 4 biological replicates for each condition). * *p* < 0.05, ** *p* < 0.01 are relative to the control condition. Statistical significance was determined by two-way ANOVA followed by Tukey’s multiple comparisons test.

Based on their daily therapeutic doses, the peak plasma concentrations of canagliflozin, empagliflozin, and dapagliflozin in patients are approximately 10 µM, 1 µM, and 0.5 µM, respectively, where the maximal percentage of the drug is bound to protein (Supplemental Figure S1) [4,39,40,41,42,43]. To reflect this pharmacologically relevant range, we tested each compound at experimental concentrations of 1, 3, and 10 µM. We first assessed whether the highest tested concentration (10 µM) induced cellular toxicity. Flow cytometry analysis confirmed that HCFs viability remained unaffected after 3 h (Supplemental Figure S2A) and 48 h (Supplemental Figure S2B) of treatment with canagliflozin, empagliflozin, or dapagliflozin, compared to control conditions (Figure 1B). Under pathological conditions, transforming growth factor-β1 (TGF-β1) promotes the conversion of fibroblasts to myofibroblasts, and stimulates cardiac fibroblast proliferation and migration [44,45,46]. TGF-β1-activated myofibroblasts are characterized by increased expression of contractile proteins such as α-smooth muscle actin (α-SMA) and exhibit enhanced secretory activity [47,48]. To assess the effects of SGLT2i on TGF-β1-induced cardiac fibroblast activation, we treated HCFs as outlined in Figure 1A. Briefly, cells were pre-incubated for 3 h with 1, 3, and 10 µM of canagliflozin, empagliflozin, or dapagliflozin, followed by 72 h of TGF-β1 stimulation. All three SGLT2i significantly decreased, in a dose-dependent fashion, the gene expression of fibrotic markers, including collagen type I (*COL1A1*) (Figure 1C), and connective tissue growth factor (CTGF) (*CCN2*) (Figure 1D). Similarly, the expression of myofibroblast markers such as periostin (*POSTN*) (Figure 1E) and α-SMA (*ACTA2*) (Figure 1F) was also suppressed in a concentration-dependent manner. Morphologically, TGF-β1-stimulated control cells adopted a large, stellate shape typical of an activated fibroblast phenotype. In contrast, HCFs pre-incubated with gliflozins appeared flattened and elongated, indicative of a quiescent phenotype (Figure 1G). We next assessed the impact of the three SGLT2i on HCFs proliferation after 48 h of treatment with 1, 3, and 10 µM of canagliflozin, empagliflozin, or dapagliflozin as shown in Figure 2A. Only canagliflozin at 10 µM significantly reduced HCF proliferation, whereas empagliflozin and dapagliflozin showed no effect at any concentration tested (Figure 2B). To evaluate fibroblast migration under SGLT2i treatment, we performed an in vitro wound healing assay (Figure 2C). A scratch was performed in confluent HCF cultures, followed by immediate treatment with 10 µM of canagliflozin, empagliflozin, or dapagliflozin. Fibroblast migration into the wound area was monitored over time. Canagliflozin at 10 µM almost completely blocked HCF migration, while empagliflozin and dapagliflozin did not produce a significant effect at the same concentration (Figure 2D,E). Together, these results suggest that while all three SGLT2i attenuate TGF-β1-induced fibroblast activation, canagliflozin exerts additional inhibitory effects on HCF proliferation and migration, indicating differential actions among SGLT2i on cardiac fibroblast behavior.

### 2.2. AMPK Activation by Canagliflozin, Empagliflozin, and Dapagliflozin in Human Cardiac Fibroblasts

**Figure 3:**
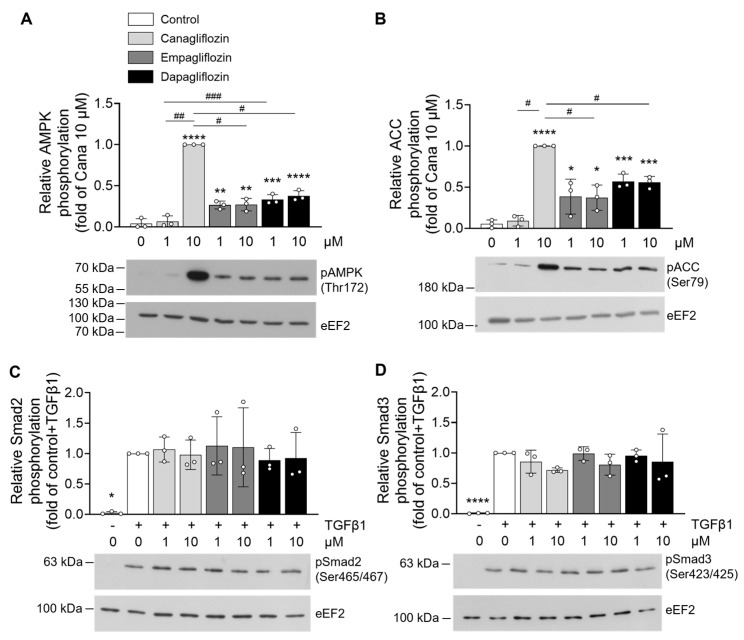
**SGLT2 inhibitors activate AMPK in human cardiac fibroblasts.** For (**A**,**B**), HCFs were treated with DMSO (vehicle) or indicated concentrations of SGLT2i for 3 h. For (**C**,**D**), TGF-β1 (10 ng/mL) was then added for 30 min. Cell lysates were submitted to Western blot analysis and probed for (**A**) phospho-AMPK(Thr172), (**B**) phospho-ACC(Ser79), (**C**) phospho-Smad2(Ser465/467), and (**D**) phospho-Smad3(Ser423/425) antibodies. eEF2 was used as a loading control. Data are expressed as mean ± SD (*n* = 3 biological replicates for each condition). Cell density for each condition: 7500 c/cm^2^ (in 6 cm diameter dishes = 21.5 cm^2^) * *p* < 0.05, ** *p* < 0.01, *** *p* < 0.001, **** *p* < 0.0001 are relative to the control condition for (**A**,**B**) and to the TGF-β1-stimulated control condition for (**C**,**D**). Statistical significance was determined by one-way ANOVA followed by Sidak’s multiple comparisons test. Statistical differences between different drug concentrations were performed by two-way ANOVA followed by multiple comparisons test. ^#^ *p* < 0.05, ^##^ *p* < 0.01, ^###^ *p* < 0.001 relative to different drug concentrations.

As previously mentioned, AMPKα1 plays a critical role in regulating cardiac fibroblast properties [38], and SGLT2i have been identified as AMPK activators in several cell types [49]. To determine whether AMPK activation also occurs in HCFs, and contributes to the regulation of fibroblast properties, we assessed the phosphorylation status of AMPK at Thr172 and its bona fide substrate, acetyl-CoA carboxylase (ACC) at Ser79, following treatment with canagliflozin, empagliflozin, or dapagliflozin at concentrations of 1 and 10 µM. Canagliflozin at 10 µM induced robust AMPK phosphorylation and associated with a marked increase in ACC phosphorylation at Ser79. In contrast, 1 µM of canagliflozin, below the expected therapeutic plasma concentration, did not activate AMPK. Both empagliflozin and dapagliflozin significantly increased AMPK phosphorylation at 1 and 10 µM, yet to a lesser extent when compared to 10 µM canagliflozin (Figure 3A,B).

Given that TGF-β1-induced fibroblast activation primarily occurs through the canonical Smad-dependent signaling pathway [50,51], we investigated SGLT2i interference with this signaling axis. Phosphorylation levels of Smad2 and Smad3 were assessed following TGF-β1 stimulation in the presence of SGLT2i. We found that none of the SGLT2i tested modified Smad2/3 phosphorylation (Figure 3C,D), suggesting that the effects of these compounds on fibroblast properties are likely mediated through an AMPK-dependent, Smad-independent mechanism.

### 2.3. AMPK-Independent and -Dependent Effects of SGLT2 Inhibitors on Human Cardiac Fibroblasts

**Figure 4:**
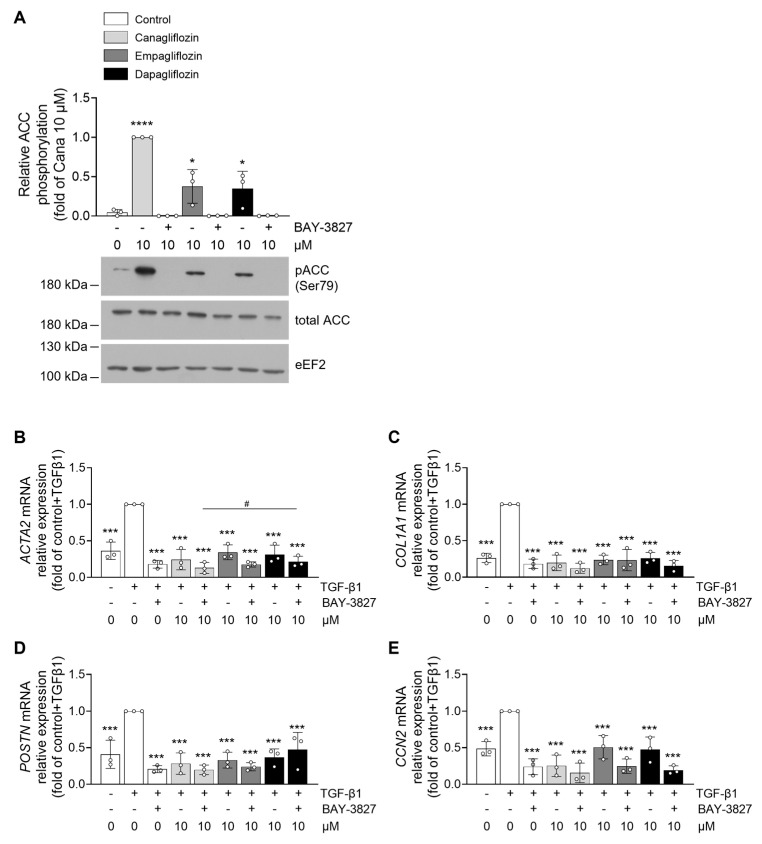
**SGLT2 inhibitors prevent cardiac fibroblast myodifferentiation in an AMPK-independent manner.** For (**A**), HCFs were incubated with the AMPK inhibitor BAY-3827 (500 nM) for 1 h prior to stimulation with DMSO (vehicle) or indicated concentrations of SGLT2i for 3 h. Cell density for each condition: 7500 c/cm^2^ (in 6 cm diameter dishes = 21.5 cm^2^). Cell lysates were submitted to Western blot analysis and probed with phospho-ACC(Ser79) and ACC antibodies. eEF2 was used as a loading control. For (**B**–**E**), HCFs were incubated with the AMPK inhibitor BAY-3827 (500 nM) for 1 h prior to stimulation with DMSO (vehicle) or indicated concentrations of SGLT2i for 72 h. Cell density for each condition: 7500 c/cm^2^ (in 10 cm diameter dishes = 60.8 cm^2^). TGF-β1 (10 ng/mL) was added 3 h after SGLT2i to induce HCF myodifferentiation. The mRNA expression levels of the myodifferentiation markers (**B**) *α-SMA*, (**C**) *COL1A1*, (**D**) *POSTN*, and (**E**) *CCN2* were measured by RT-qPCR on RNA extracts. Data are expressed as mean ± SD (*n* = 3 biological replicates for each condition). * *p* < 0.05, *** *p* < 0.001, **** *p* < 0.0001 are relative to the control condition for (**A**) and to the TGF-β1-stimulated control condition for (**B**–**E**). Statistical significance was determined by one-way ANOVA followed by Sidak’s multiple comparisons test. Statistical differences between different drug concentrations were performed by two-way ANOVA followed by multiple comparisons test. ^#^ *p* < 0.05 relative to different drug concentrations.

**Figure 5:**
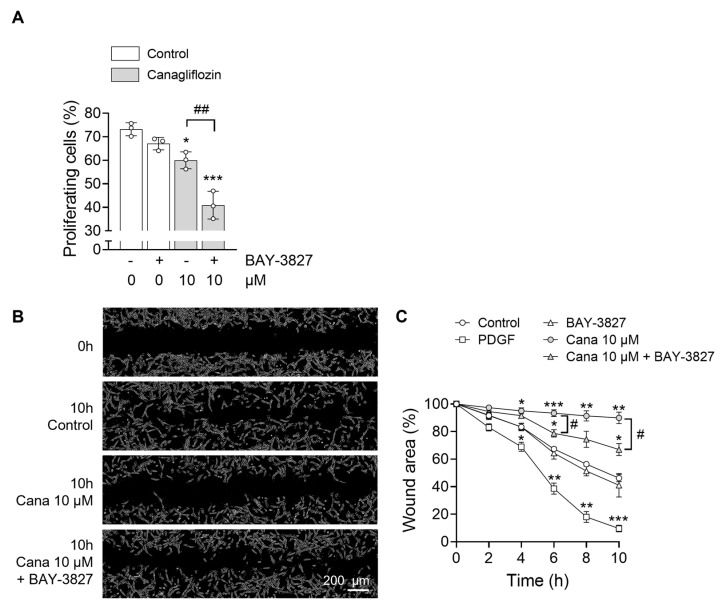
**Canagliflozin reduces cardiac fibroblast proliferation independently of AMPK but relies on AMPK to suppress migration.** For (**A**), HCFs were incubated with the AMPK inhibitor BAY-3827 (500 nM) for 1 h prior to stimulation with DMSO (vehicle) or canagliflozin 10 µM in a medium containing 1% growth factors for 48 h. Cell density for each condition: 5000 c/cm^2^ (in 6 cm diameter dishes = 21.5 cm^2^). Proliferation was quantified by flow cytometry using 5-ethynyl-2′-deoxyuridine (EdU). Percentage of proliferating (EdU-positive) cells. For (**B**,**C**), a scratch was made in the cell layer and HCFs were incubated with the AMPK inhibitor BAY-3827 (500 nM) for 1 h prior to stimulation with DMSO (vehicle), PDGF (40 ng/mL) used as a positive control, or canagliflozin 10 µM. Microscopic pictures were taken at baseline and after 2, 4, 6, 8 and 10 h to assess the refilling of the wound by fibroblasts. (**B**) Representative images (scale 200 µM) and (**C**) quantification of wound recolonization. Cell density for each condition: 10 000 c/cm2 (in 3.5 cm diameter dishes = 9.4 cm^2^). Data are expressed as mean ± SD (*n* = 3 biological replicates for each condition). * *p* < 0.05, ** *p* < 0.01, *** *p* < 0.001 are relative to the control condition. ^#^ *p* < 0.05, ^##^ *p* < 0.01 are relative to the canagliflozin 10 µM condition. Statistical significance was determined by one-way ANOVA followed by Sidak’s multiple comparisons test for (**A**) and by two-way ANOVA followed by Tukey’s multiple comparisons test for (**C**).

To determine whether the effects of SGLT2i on cardiac fibroblast properties are dependent on AMPK signaling, we sought to inhibit AMPK activity. However, transfection of HCFs with AMPK-specific small interfering RNA (siRNA) was not feasible, as it disrupted the myodifferentiation process. As an alternative, we employed BAY-3827, a recently developed and highly specific AMPK inhibitor. HCFs were pre-incubated with BAY-3827 (500 nM) for 1 h prior to stimulation with 10 µM canagliflozin, empagliflozin, or dapagliflozin. AMPK activation was assessed by measuring the phosphorylation of its downstream substrate, ACC at Ser79, rather than AMPK phosphorylation itself, as BAY-3827 has been shown to paradoxically increase Thr172 phosphorylation [52]. As expected, BAY-3827 effectively abolished ACC phosphorylation, confirming inhibition of AMPK signaling (Figure 4A). To determine whether the effects of SGLT2i on TGF-β1-induced myodifferentiation were AMPK-dependent, we repeated the experiment depicted in Figure 1A on HCFs pretreated or not with BAY-3827. Notably, the ability of canagliflozin, empagliflozin, or dapagliflozin to suppress myofibroblast gene expression was preserved despite AMPK inhibition. RT-qPCR analysis revealed that mRNA levels of gene expression of ACTA2 (Figure 4B), COL1A1 (Figure 4C), POSTN (Figure 4D), and CCN2 (Figure 4E) remained significantly suppressed by SGLT2i, even in the presence of BAY-3827. Interestingly, BAY-3827 alone also significantly reduced the expression of these myofibroblast markers, suggesting that TGF-β1-induced myodifferentiation is, at least in part, dependent on AMPK activation. Given that canagliflozin demonstrated the strongest AMPK activation among the tested SGLT2i, it was selected for subsequent experiments assessing cardiac fibroblast proliferation and migration. Pretreatment with BAY-3827 did not prevent 10 µM canagliflozin from reducing HCF proliferation (Figure 5A). While BAY-3827 alone had no effects on proliferation, its combination with canagliflozin led to an additive anti-proliferative effect (Figure 5A). Lastly, we evaluated the impact of AMPK inhibition on canagliflozin-mediated regulation of fibroblast migration using the wound healing assay. In this setting, BAY-3827 partially reversed the anti-migratory effect of canagliflozin (Figure 5B,C), indicating that the inhibition of fibroblast migration by canagliflozin is partially mediated by an AMPK-dependent mechanism.

## 3. Discussion

This study demonstrates that canagliflozin, empagliflozin, and dapagliflozin, at clinically relevant concentrations, differentially regulate cardiac fibroblast properties through both AMPK-dependent and -independent mechanisms. While all three SGLT2i effectively prevent TGF-β1-induced myodifferentiation, only canagliflozin reduces fibroblast proliferation and migration. These anti-differentiation effects are AMPK-independent, whereas canagliflozin’s anti-migration action is at least partially AMPK-dependent. To our knowledge, this is the first direct comparison of these three SGLT2i in HCFs.

SGLT2i emerged as promising and effective drugs for the treatment of patients with heart failure, both with preserved or reduced ejection fraction, regardless of the presence of diabetes [53,54]. This cardioprotective effect of SGLT2i is particularly intriguing given that SGLT2 expression is not detectable in the healthy heart [13,14,15,55]. Over the past decades, several off-target mechanisms have been proposed, including inhibition of NHE-1, SGLT1, and Nav1.5 channel, contributing to reduce intracellular sodium and calcium levels, decrease reactive oxygen species (ROS) production, and attenuate inflammation in cardiac cells [18,22,35,56,57].

Cardiac fibroblasts play a key role in extracellular matrix remodeling following injury. Their proliferation and migration are essential for post-infarction repair, whereas persistent myofibroblast activation, often driven by chronic TGF-β1 signaling, contributes to maladaptive interstitial fibrosis and diastolic dysfunction [31]. Given the strong association between fibrosis and adverse outcomes in heart failure with both preserved and reduced ejection fraction, targeting fibroblast activity is of high therapeutic interest [31,58,59]. Although SGLT2i have shown anti-fibrotic effects in rodent models of heart failure [33,35,60,61,62,63,64], direct comparative studies among canagliflozin, empagliflozin, and dapagliflozin across fibroblast functions in human cells are lacking. Our data show that canagliflozin uniquely suppresses the proliferation and migration of HCFs, reinforcing the idea that SGLT2i effects may be compound-specific rather than class-wide. These findings are consistent with prior observations where canagliflozin, but not other gliflozins, reduced proliferation in endothelial and cancer cells [25,65].

AMPK has emerged as a key regulator of fibroblast activation [38], and SGLT2i are known to activate AMPK in several cell types [49]. We observed that canagliflozin induced stronger AMPK and ACC phosphorylation in HCFs than empagliflozin or dapagliflozin, consistent with previous studies [25,26,66]. This effect has been linked to mitochondrial inhibition, a unique property of canagliflozin. However, the exact mechanisms through which SGLT2i activate AMPK remain unclear. Importantly, our findings reveal that the effects of SGLT2i on cardiac fibroblast properties persist in AMPK-inhibited cells, with the exception of canagliflozin-induced suppression of fibroblast migration, which was partially reversed by BAY-3827. Using the specific AMPK inhibitor BAY-3827, we found that AMPK inhibition did not prevent SGLT2i from reducing fibrotic gene expression, confirming an AMPK-independent mechanism. However, BAY-3827 partially reversed the anti-migratory effect of canagliflozin, indicating that AMPK contributes to certain fibroblast-modulatory actions.

Smad2/3 phosphorylation, a hallmark of canonical TGF-β1 signaling, remained unchanged with SGLT2i treatment, suggesting that SGLT2i do not interfere with this pathway. Although we have shown that in human cardiac fibroblasts, phosphorylation of ERK1/2 is not affected by any of the tested gliflozins (Supplemental Figure S3), in different models, SGLT2i inhibit MAPK pathways, including ERK1/2, JNK, and p38, in a model of transverse aortic constriction [67], as well as in vitro cardiac and vascular cells [68,69], which may also modulate fibroblast behavior. The JAK2-STAT3 pathway, another key profibrotic signaling, is activated in cardiac fibroblasts during fibrosis [70,71]. Interestingly, empagliflozin has been shown to inhibit this pathway in macrophages [72,73], raising the possibility of similar effects in fibroblasts, though this remains to be confirmed.

SGLT1, initially proposed as an off-target of SGLT2i [74], appears to be expressed in the heart only as a truncated non-functional isoform [75]. Our findings support this, showing negligible expression of both SGLT1 and SGLT2 in HCFs. Interestingly, we previously demonstrated that the only functional SGLT expressed in the human, murine, and rodent heart is the myo-inositol and sodium-coupled cotransporter SMIT1 [13] which controls cardiac fibroblasts properties in vitro [76]. Its role in fibrosis, and the possible impact of SGLT2i on its activity, remains speculative and warrants further exploration.

Our study has several limitations. First, SGLT2i were administered prior to TGF-β1 stimulation, which may not reflect the clinical scenario where fibrosis is already established. Nevertheless, this approach allowed us to investigate their potential preventive effects. Second, although BAY-3827 is a more selective AMPK inhibitor than compound C, off-target effects cannot be completely excluded. Using siRNA was not feasible due to its interference with fibroblast differentiation, and AMPKα1-knockout fibroblasts were unsuitable due to spontaneous differentiation in vitro [77]. In addition, the fibroblasts used in this study were isolated from adult human hearts, adding an important translational value. However, limited information about donor clinical backgrounds introduces possible heterogeneity in cellular responses. Another constraint is that we focused exclusively on TGF-β1-induced myodifferentiation, whereas in vivo cardiac fibrosis involves multiple profibrotic mediators and dynamic cellular interactions. Furthermore, while fibroblasts are the primary source of extracellular matrix proteins, other cell types such as immune cells, vascular cells, and cardiomyocytes also play a major role in the development of fibrosis [31]. The potential impact of SGLT2i on these other cell types remains to be explored. Finally, while our data show that SGLT2 expression is barely detectable in cardiac fibroblasts, we did not directly investigate specific off-target pathways. For instance, we did not assess the role of NHE-1 inhibition using selective pharmacological inhibitors or siRNA. Future studies should therefore address these alternative targets to clarify the full spectrum of SGLT2i mechanisms in cardiac fibroblast biology.

In summary, our findings reveal that canagliflozin, empagliflozin, and dapagliflozin exert distinct effects on HCF function. In particular, canagliflozin exerts broader anti-fibrotic actions, suppressing differentiation, proliferation, and migration partially via AMPK activation. These data support the notion that the cellular effects of SGLT2i may be compound-specific rather than class-wide. Future studies in animal models and human tissues are needed to determine how these differences translate to therapeutic outcomes in cardiac fibrosis and heart failure.

## 4. Materials and Methods

### 4.1. Materials and Reagents

Canagliflozin (#HY-10451), empagliflozin (#HY-15409), dapagliflozin (#HY-10450), and BAY-3827 (#HY-112083) were obtained from MedChemExpress (Monmouth Junction, NJ, USA). Recombinant human TGF-β1 protein (active) (#ab50036) and platelet-derived growth factor (PDGF) protein (active) (#ab259425) were purchased from Abcam (Cambridge, UK). The antibodies employed were phospho-ACC(Ser79) (#3661; Cell Signaling Technology, Danvers, MA, USA), ACC (#3676; Cell Signaling Technology), phospho-AMPK(Thr172) (#2535; Cell Signaling Technology), AMPKα1 (#MA5-15815; Thermo Fisher Scientific, Waltham, MA, USA), phospho-Smad2(Ser465/467) (#3108; Cell Signaling Technology), phospho-Smad3(Ser423/425) (#ab52903; Abcam), eEF2 (#PA5-17794; Thermo Fisher Scientific), and secondary horseradish peroxidase (HRP)-conjugated anti-rabbit IgG (#RABHRP1; Sigma-Aldrich, Overijse, Belgium).

### 4.2. Primary Human Cardiac Fibroblast Culture

HCFs were purchased from ScienCell Research Laboratories (Carlsbad, CA, USA, #6300). Clinical information on human donors was not provided. HCFs were negative for HIV-1, HBV, HCV, mycoplasma, bacteria, yeast, and fungi. They were cultured according to the manufacturer’s recommendations using HCF basal medium (#315-500, Tebu-Bio, Le Perray-en-Yvelines, France) containing 1% penicillin-streptomycin and 10% fibroblast growth supplement (#316-GS, Tebu-Bio), at 37 °C and 5% CO_2_ in a humidified incubator. The cells were divided when they reached 90% confluence and used up to a maximum passage number of three for myodifferentiation assessment and seven for other experiments. Cells were starved of growth factors for 2 h (toxicity test, myodifferentiation assessment, and Western blot) or 24 h (proliferation and migration assessment) prior to treatment.

### 4.3. Toxicity Test

HCFs were stimulated with indicated concentrations of SGLT2i for 3 or 48 h. The percentage of viable cells (corresponding to Annexin V-negative/propidium iodide-negative cells) was measured by flow cytometry (BD Biosciences FACSCanto^TM^, San Jose, CA, USA), using the Annexin V-FITC Apoptosis Detection Kit (#APOAF, Sigma-Aldrich) according to the manufacturer’s recommendations.

### 4.4. Cell Proliferation Assay

HCFs were stimulated with indicated concentrations of SGLT2i in a medium containing 1% growth factors for 48 h. 5-ethynyl-2′-deoxyuridine (EdU) was added at the time of stimulation. Proliferation was measured by flow cytometry (BD Biosciences FACSCanto^TM^) using the Click-iT^TM^ EdU Alexa Fluor^TM^ 488 Flow Cytometry Assay Kit (#C10425, Thermo Fisher Scientific) according to the manufacturer’s recommendations.

### 4.5. Wound Healing Scratch Assay

HCFs were grown to 90% confluence before scratching the cell layer with a sterile pipette tip. Cells were stimulated with indicated concentrations of SGLT2i or PDGF used as positive control. The whole experiment was performed in the absence of serum. Images were obtained after 2, 4, 6, 8, and 10 h using a microscope (Olympus TH4-200, Japan) coupled to a camera to evaluate wound recolonization by fibroblasts. Image analysis and quantification of free area filling were performed using TScratch software (CSE Laboratory, ETH Zürich, Switzerland, http://www.cse-lab.ethz.ch/software.html (accessed on 14 October 2025)).

### 4.6. Myodifferentiation Assay

HCFs were serum deprived for 2 h before stimulation with SGLT2i. After 3 h of SGLT2i treatment, HCFs myodifferentiation was induced by TGF-β1 (10 ng/mL) for 72 h. The whole experiment was performed in the absence of serum. Myodifferentiation was measured via mRNA expression levels of fibrotic markers (*COL1A1*, *CCN2, POSTN*, and *α-SMA*).

### 4.7. RNA Extraction and mRNA Expression

Total RNA was isolated from HCFs using the RNeasy mini-kit (#74106, Qiagen, Venlo, The Netherlands) after cells lysis with TriPure™ Isolation Reagent (#11667165001, Sigma-Aldrich) and samples were treated with DNAse (#79256, Qiagen) according to the manufacturer’s instructions. RNA was quantified using a NanoDrop^TM^ spectrophotometer (Thermo Fisher Scientific). 1 µg of RNA was reverse-transcribed using the iScript^TM^ cDNA Synthesis Kit (#1708891, Bio-Rad, Hercules, CA, USA), and real-time quantitative PCR (RT-qPCR) using the qPCR Core Kit for SYBR Green I-No ROX (#RT-SN10-05NR, Eurogentec, Seraing, Belgium) was used for mRNA expression analysis. Reactions were performed on an IQ5 apparatus (Bio-Rad). RPL32 was used as a housekeeping gene for mRNA measurement. Primer sequences are presented in Supplemental Table S1.

### 4.8. Western Blot Analysis

HCFs were lysed in cold buffer containing 50 mM Tris-HCl pH 7.5, 1 mM EDTA, 1 mM EGTA, 0.27 M sucrose, 1% (*w*/*v*) Triton X-100, 20 mM glycerol-2-phosphate disodium, 50 mM NaF, 5 mM Na_4_P_2_O_7_^.^10H_2_O, 1 mM DTT, and 1× Halt^TM^ protease and phosphatase inhibitor cocktail (#78446, Thermo Fisher Scientific, Waltham, MA, USA). The lysates were centrifuged at 15,000× *g* at 4 °C for 10 min and the protein content of the supernatant was measured by the Bradford method using bovine serum albumin (BSA) as a reference. An equal amount of proteins was resuspended in a modified Laemmli’s buffer containing 50 mM Tris pH 6.8, 10% glycerol, 2% sodium dodecyl sulfate (SDS), 0.5 mM EDTA, 23 mM DTT, and 0.01% bromophenol blue. Proteins were separated by SDS-polyacrylamide gel electrophoresis and transferred to polyvinylidene difluoride membranes. Membranes were blocked with Tris-buffered saline (TBS)/0.01% Tween 20/5% BSA and probed overnight at 4 °C with the corresponding primary antibody at appropriate dilution: phospho-ACC (1:1000), ACC (1:1000), phospho-AMPK (1:1000), α1AMPK (1:1000), phospho-Smad2 (1:1000), phospho-Smad3 (1:1000), and eEF2 (1:1000). After incubation with HRP-conjugated IgG secondary antibody (1:20,000) for 1 h at room temperature, proteins were detected by chemiluminescence. Quantification was performed using Image J software (version 1.8, National Institutes of Health, Bethesda, MD, USA). eEF2 was used as a loading control. Band intensities were normalized relative to the loading controls on the same gel (uncropped original blots are present in Supplemental Figure S4).

### 4.9. Statistics

Statistical analysis was performed using GraphPad Prism 10.1.0. All data herein are presented as the mean ± SEM. Statistical significance was determined by one-way ANOVA followed by Sidak’s multiple comparisons test when more than two groups were compared. Analysis was performed using two-way ANOVA with Tukey’s multiple-comparison test, as indicated, with differences noted as statistically significant when *p* ≤ 0.05. Grubbs’s test was used to exclude statistical outliers.

## Data Availability

The authors declare that all the data supporting the findings of this study are available within the paper and its Supplementary Data.

## Supplementary Materials

The following supporting information can be downloaded at: https://www.mdpi.com/article/10.3390/ijms262010098/s1.

## Abbreviations

The following abbreviations are used in this manuscript:

ACC	Acetyl-CoA carboxylase
AMPK	AMP-activated protein kinase
CTGF	Connective tissue growth factor
DMSO	Dimethyl sulfoxide
EdU	5-ethynyl-2′-deoxyuridine
eEF2	Eukaryotic elongation factor 2
HCF	Human cardiac fibroblast
NHE-1	Na^+^/H^+^ exchanger 1
PDGF	Platelet-derived growth factor
RPL32	Ribosomal protein L32
SGLT	Sodium-glucose cotransporter
SGLT2i	Sodium-glucose cotransporter 2 inhibitors
TGF-β	Transforming growth factor-β
