# Supplementary material for: Comparison of the Effects of Sodium-Glucose Cotransporter 2 Inhibitors on Cardiac Fibroblast Properties
Source: Int J Mol Sci. 2025 Oct 16;26(20):10098. doi: 10.3390/ijms262010098 (PMC12564173; doi:10.3390/ijms262010098)
Supplement: Supplementary file 1 [file ijms-26-10098-s001.zip › ijms-3907908-supplementary.pdf]

Supplemental File

Comparison of the Effects of Sodium-Glucose Cotransporter 2 Inhibitors on Cardiac Fibroblast Properties

Claire Baufays [1,2], Julien Cumps [1], Cécile Dufeys [1], Audrey Ginion [1], Luc Bertrand [1], Sandrine Horman [1], Christophe Beauloye [1,2,3,*,†] and Alice Marino [1,*,†]

[1] Pôle de Recherche Cardiovasculaire, Institut de Recherche Expérimentale et Clinique, Université Catholique de Louvain, 1200 Brussels, Belgium; claire.baufays@uclouvain.be (C.B.); julien.cumps@uclouvain.be (J.C.); cecile.dufeys@uclouvain.be (C.D.); audrey.ginion@uclouvain.be (A.G.); luc.bertrand@uclouvain.be (L.B.); sandrine.horman@uclouvain.be (S.H.)
[2] Division of Cardiology, Cliniques Universitaires Saint-Luc, 1200 Brussels, Belgium
[3] Division of Cardiovascular Intensive Care, Cliniques Universitaires Saint-Luc, 1200 Brussels, Belgium
***** Correspondence: christophe.beauloye@uclouvain.be (C.B.); alice.marino@uclouvain.be (A.M.)
[†] Contributed equally and are joint last authors.

Table S1. List of primers sequences used in the study.

Gene	Species	Sense	Sequence 5' → 3'	T (°C)	Amplicon Size	Accession number
RPL32	Human	Forward	AGGCATTGACAACAGGGTTC	62.5	160	NM_000994
		Reverse	GTTGCACATCAGCAGCACTT			
α-SMA	Human	Forward	ACTGGGACGACATGGAAAAG	62.5	208	NM_0011141945
		Reverse	GCGTCCAGAGGCATAGAGAG			
COL1A1	Human	Forward	GGACACAGAGGTTTCAGTGGT	63.3	100	NM_000088
		Reverse	GCACCATCATTTCCACGAGC			
POSTN	Human	Forward	AGACTGCTTCAGGGAGACACA C	62.5	96	NM_006475
		Reverse	TCACTGAGAACGACCTTCCCT T			
CTGF	Human	Forward	CCGTACTCCCAAAATCTCCA	60	211	NM_001901
		Reverse	GTAATGGCAGGCACAGGTCT			

Canagliflozin	Empagliflozin	Dapagliflozin
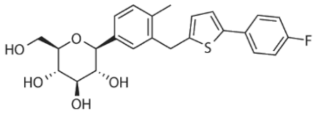	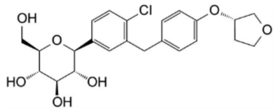	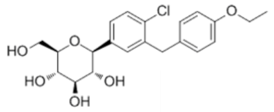
MW: 444.14 g/mol	MW: 450.91 g/mol	MW: 408.88 g/mol
SGLT1 inhibition IC_{50}: 710 nM	SGLT1 inhibition IC_{50}: 8300 nM	SGLT1 inhibition IC_{50}: 1400 nM
SGLT2 inhibition IC_{50}: 2.7 nM	SGLT2 inhibition IC_{50}: 3.1 nM	SGLT2 inhibition IC_{50}: 1.2 nM
$T_{1/2}$: 13.1 h	$T_{1/2}$: 12.4 h	$T_{1/2}$: 12.9 h
Fraction bound to protein: 99%	Fraction bound to protein: 86%	Fraction bound to protein: 91%

Figure S1. Comparative illustration and physicochemical properties of canagliflozin, empagliflozin and dapagliflozin. MW: molecular weight; T1/2: time of half-life; IC50: half-maximal inhibitory concentration [44].

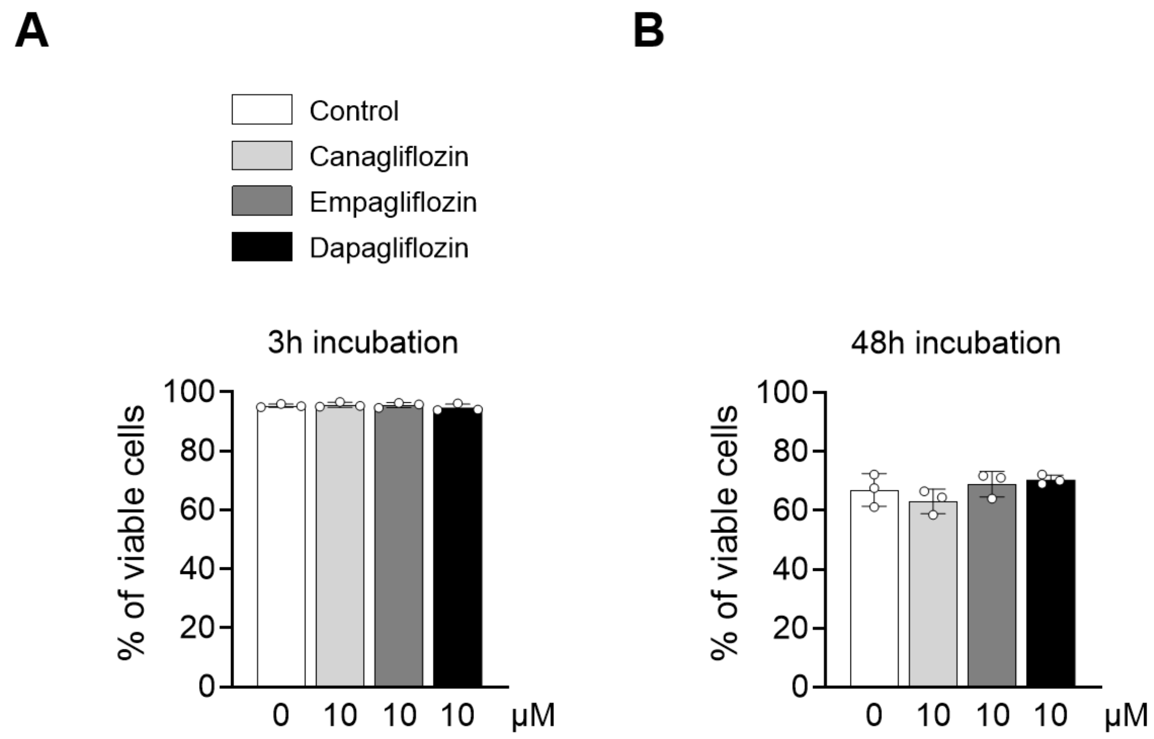

Figure S2. Canagliflozin, empagliflozin or dapagliflozin used at 10 μM concentration are non-toxic to human cardiac fibroblasts over short or long incubation periods. Percentage of viable cells was determined by flow cytometry using Annexin V-FITC and propidium iodide staining following **(A)** 3 or **(B)** 48 hours stimulation with DMSO (vehicle) or 10 μM canagliflozin, empagliflozin or dapagliflozin. Data are expressed as mean ± SD (n = 3 biological replicates for each condition). Statistical significance was determined by one-way ANOVA followed by Sidak's multiple comparisons test.

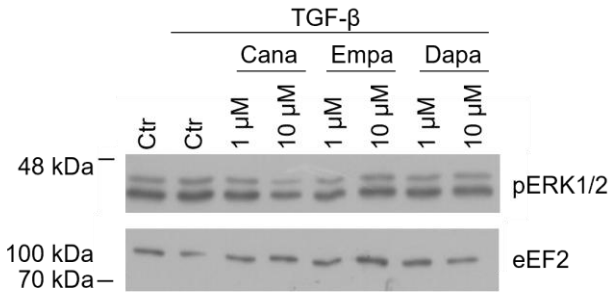

Figure S3. SGLT2 inhibitors do not impact non-canonical HCF myodifferentiation pathways. HCFs were treated with DMSO (Ctr) or indicated concentrations of SGLT2i for 3 hours. TGF-β1 (10 ng/mL) was then added for 30 min. Cell lysates were submitted to western blot analysis and probed with (A) phospho-ERK1/2. eEF2 was used as loading control.

Figure 3. SGLT2 inhibitors activate AMPK in human cardiac fibroblasts. . Cell lysates were submitted to Western blot analysis and probed for **(A)** phospho-AMPK(Thr172) **(B)** phospho-ACC(Ser79), **(C)** phospho-Smad2(Ser465/467), and **(D)** phospho-Smad3(Ser423/425) antibodies. eEF2 was used as a loading control. Data are expressed as mean ± SD (n = 3 biological replicates for each condition).

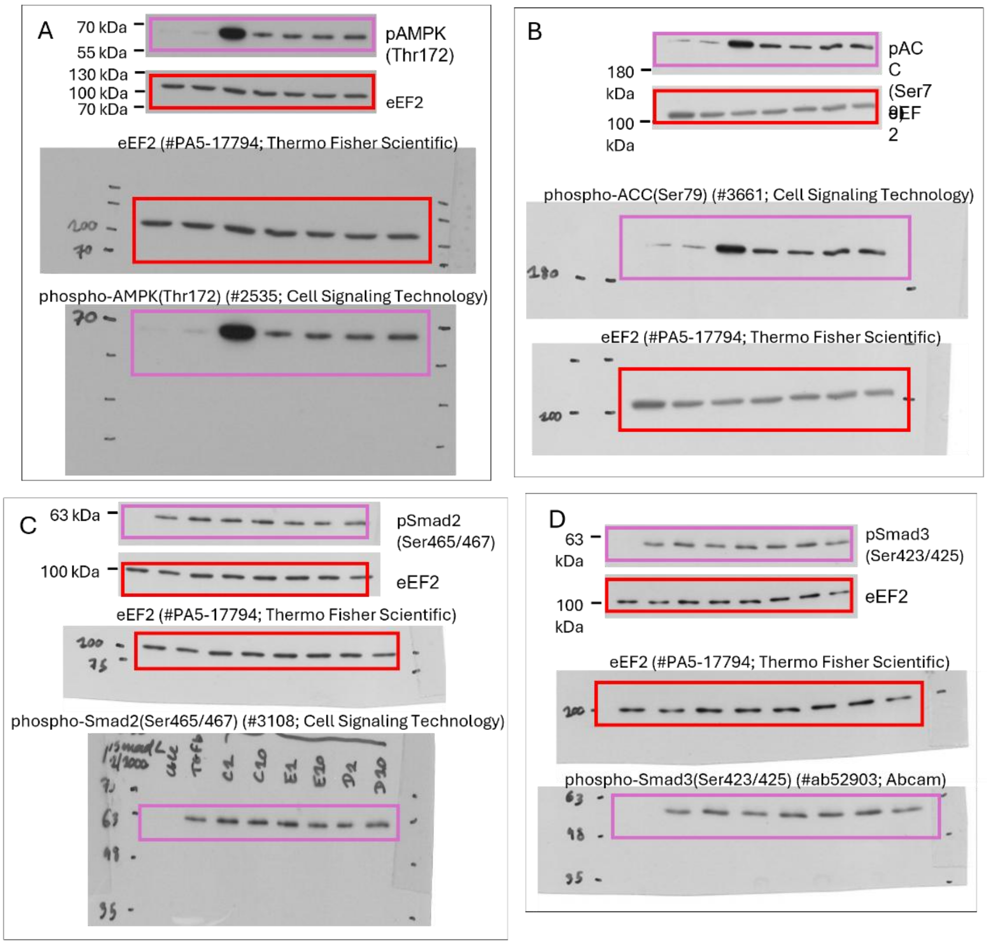

Figure 4. SGLT2 inhibitors prevent cardiac fibroblast myodifferentiation in an AMPK-independent manner. For **(A).** Cell lysates were submitted to Western blot analysis and probed with phospho-ACC(Ser79) antibodies. eEF2 was used as a loading control.

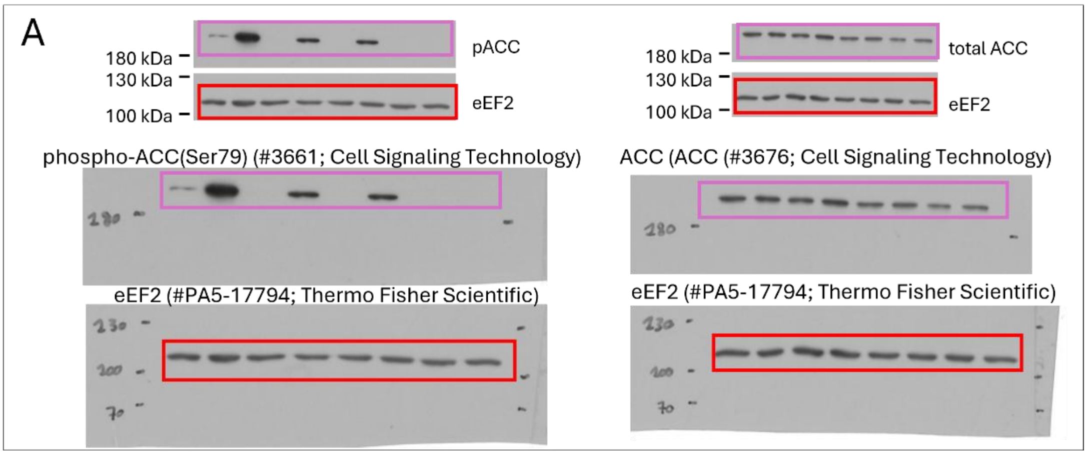

Figure S4. Uncropped gels of Figure 3 and Figure 4 of the main manuscript.
